# Sutureless aortic valve replacement in pure aortic regurgitation: expanding the indications

**DOI:** 10.1186/s13019-022-01959-8

**Published:** 2022-08-22

**Authors:** Alina Zubarevich, Arian Arjomandi Rad, Lukman Amanov, Marcin Szczechowicz, Anja Osswald, Saeed Torabi, Bastian Schmack, Arjang Ruhparwar, Alexander Weymann

**Affiliations:** 1 Department of Thoracic and Cardiovascular Surgery, West German Heart and Vascular Center, University of Duisburg-Essen, Hufelandstraße 55, 45122 Essen, Germany; 2 Clinical Academic Graduate School, University of Oxford, Oxford, UK; 3 Department of Anesthesiology, University Hospital Cologne, Cologne, Germany

**Keywords:** SU-AVR, Pure aortic regurgitation, Perceval

## Abstract

**Background:**

In the era of transcatheter methods, patients presenting with a pure aortic regurgitation (AR) are not considered eligible for transcatheter treatment and therefore require another less invasive surgical option. We sought to review our experience with sutureless aortic valve replacement (SU-AVR) in patients presenting with symptomatic pure AR, which until now is a contraindication for implementation of sutureless valve prostheses in Europe.

**Methods:**

Between April 2018 and June 2021, 80 consecutive patients underwent a SU-AVR for various indications at our institution. We analyzed the outcomes and postoperative complications of 12 patients presenting with a pure severe AR undergoing SU-AVR using Perceval (Corcym).

**Results:**

The mean age of the patients was 67 ± 9.1 years old. All patients presented with symptomatic pure AR. Patients presented with multiple comorbidities as reflected by the mean EuroSCORE-II of 3.6 ± 2.6%. Six patients (50%) underwent a concomitant CABG procedure. The mean operating- and cross clamp time was 127.25 ± 45.9 and 40.33 ± 17.3 min respectively. All isolated SU-AVR were performed via J-sternotomy or right anterolateral thoracotomy. There were no cases of device dislocation. No patients presented with a paravalvular leakage. We observed excellent mean postoperative pressure gradient at follow-up 5.7 ± 1.5 mmHg.

**Conclusions:**

Our experience with SU-AVR shows the feasibility of sutureless technologies in the aortic valve surgery due to pure AR. Besides the great technical success and excellent hemodynamics, SU-AVR in severe AR offers a great opportunity of reducing the invasivity of the surgical procedure and potentially reducing hospital cost without compromising the postoperative outcomes and in-hospital length of stay.

## Background

Until recently, surgical aortic valve replacement has been the most common therapeutic approach in patients presenting with severe aortic valve pathologies [1]. Over the last years, the treatment options for severe aortic valve disease have been drastically expanded towards the transcatheter methods, which have currently become the treatment of choice even in intermediate- and low-risk patients presenting with severe stenotic aortic valve disease [2]. At the same time, the conventional surgical methods of treatment are becoming more minimally invasive to keep up with the transcatheter treatment options. Especially patients presenting with the symptomatic pure severe aortic regurgitation benefit from the minimally invasive surgical methods, as in the most cases these patients are not eligible for transcatheter treatment.

Sutureless aortic valve replacement (SU-AVR) has been introduced into the cardiac surgery almost half a decade ago to simplify the surgical procedure and reduce the cardiopulmonary bypass (CPB)- and cross-clamp time, which are commonly known to be independent predictors of mortality and morbidity [3–5]. Since then, the implantation techniques of sutureless aortic valve prostheses have evolved and SU-AVR has proven to be a safe and effective treatment option in various indications and combined valve procedures [6–10].

In this study we sought to review our experience with SU-AVR in patients presenting with symptomatic pure severe aortic valve regurgitation, which until now has been considered a contraindication for implementation of sutureless aortic valve prostheses.

## Materials and methods

### Study design and population

Between April 2018 and June 2021, 80 consecutive patients underwent a SU-AVR for various indications at our institution. Of those, 12 patients underwent a SU-AVR due to pure severe aortic regurgitation. We analyzed the outcomes and postoperative complications in patients undergoing SU-SAVR using Perceval (Corcym) due to pure severe aortic regurgitation. Patients presenting with infective endocarditis and those in whom a concomitant valve procedure was required have been excluded from the study. Data were obtained from the institutional database that includes detailed information on patients’ demographics, baseline clinical characteristics, and their laboratory and hemodynamic parameters, as well as intraoperative variables and postoperative outcomes. Patients were followed up based on information available in their electronic medical records, as well as through telephone interviews. This study and its’ methods conform to the guidelines of the 1975 Declaration of Helsinki as reflected in a prior approval by the institutional Ethical Committee of University Duisburg- Essen (Registration number 21-10349-BO) and the patients’ individual written informed consent has been waived.

### Operative techniques

Prior to all surgical procedures involving valve surgery we perform echocardiography and angiography for preoperative planning.

For SU-AVR, the heart was accessed via median sternotomy, J-sternotomy or right anterolateral thoracotomy (RALT). Cardiopulmonary bypass (CPB) was initiated with a direct cannulation of the ascending aorta and cannulation of the right atrium. Moderate hypothermic cardiac arrest at 35 °C was performed in all procedures. Myocardial protection was achieved with cold crystalloid cardioplegia. The aortic valve was exposed and excised through an oblique aortotomy. The implantation of sutureless Perceval prostheses was performed using the “Snugger-method” as described previously, and the aortotomy was closed with a 4.0 prolene suture. After assessment of the valve performance and careful de-airing, the patient was weaned from the CPB [6].

The closure of patent foramen ovale (PFO) was performed on CBP on the beating heart and coronary arterial bypass grafting (CABG) was performed on the arrested heart prior to the SU-AVR. Proximal coronary anastomoses were performed on the arrested heart to avoid additional manipulation of the aorta after the sutureless valve prosthesis had been implanted.

### Outcomes and definitions

The primary endpoints were technical success of the procedure and 30-day- and follow-up mortality. The secondary endpoint was the development of any complications according to Valve Academic Research Consortium-2 [11].

### Statistical analysis

Statistical analysis was performed using IBM SPSS version 27 (IBM Corp., Chicago, IL, USA) and R software v.3.4.3 (R Foundation for Statistical Computing, Vienna, Austria). Data were tested for normality using the Shapiro–Wilk test. Continuous variables were expressed as medians (interquartile range, IQR) or as mean ± standard deviation. Categorical variables were expressed as frequencies and percentages.

## Results

### Baseline characteristics

The mean age of the patients was 67 ± 9.1 years old (Table 1). All patients presented with symptomatic pure severe aortic regurgitation. Patients presented with multiple comorbidities as reflected by the median logistic EuroSCORE of 6.2% (IQR 1.8–13.3) and a mean EuroSCORE-II of 3.6 ± 2.6%. Eight patients (66.7%) were suffering from coronary artery disease. The mean left ventricular ejection fraction was slightly reduced at 53.3 ± 7.8%.

**Table 1:** Baseline characteristics

Characteristics	n (%)
Female gender	4 (33.3)
Age, years	67 ± 9.1
BMI, kg/qm	26.1 ± 4.5
Arterial hypertension	12 (100)
Hyperlipoproteinemia	6 (50)
Coronary arterial disease	8 (66.7)
Peripheral arterial disease	0
Chronic kidney injury	3 (25)
Dialysis	1 (8.3)
Previous myocardial infarction	2 (16.7)
Chronic obstructive pulmonary disease	2 (16.7)
Impaired LVF	3 (25)
Ejection fraction,%	53.3 ± 7.8
AR ≥ II	12 (100)
NYHA-class	
I	1 (8.3)
II	6 (50)
III	5 (41.7)
Diabetes mellitus	4 (33.3)
EuroSCORE II	3.6 ± 2.6

BMI, Body Mass Index; LVF, left ventricular function; AR, aortic regurgitation; NYHA, New York Heart Association

### Intraoperative characteristics

Out of all the patients, eight (66.7%) underwent an elective procedure (Table 2). Six patients (50%) underwent a concomitant CABG procedure. Of the whole cohort, two patients (16.7%) had previously undergone a cardiac procedure via median sternotomy. The mean operating time was 127.25 ± 45.9 min and the mean cross-clamp time was 40.33 ± 17.3. All isolated SU-AVR were performed via J-sternotomy or RALT.

**Table 2:** Intraoperative characteristics

Characteristics	n (%)
Redo	2 (16.7)
Elective	8 (66.7)
Urgent	2 (16.7)
Emergernt	2 (16.7)
Prosthesis size	
S	3 (25)
M	1 (8.3)
L	4 (33.3)
XL	4 (33.3)
Concomitant procedure	7 (58.3)
CABG	6 (50)
PFO closure	1 (8.3)
Operating time, min	127.25 ± 45.9
Cross-clamp time, min	40.33 ± 17.3

CABG, coronary artery bypass grafting; PFO, patent foramen ovale

### Postoperative characteristics and survival

The mean follow-up time was 350.8 ± 177.1 days (Table 3). Technical success was achieved in all patients. There were no cases of intraoperative- or postoperative device dislocation. We did not observe any cases of stroke in our cohort. No patients required a postoperative permanent pacemaker implantation or presented with a paravalvular leakage. Additionally, we observed excellent mean postoperative pressure gradient at follow-up 5.7 ± 1.5 mmHg. We also report no deaths at follow-up and no cases of early infective endocarditis.

**Table 3:** Postoperative outcomes

Characteristics	n (%)
ICU-stay, days	2.0 (IQR 1.0–30)
In-hospital stay, days	11 ± 2.6
Stroke	0
Pacemaker implantation	0
New onset dialysis	1 (8.3)
Re-thoracotomy	1 (8.3)
Transvalvular mean gradient at follow-up, mmHg	5.7 ± 1.5
Device technical success	12 (100%)
Paravalvular leakage	0
Device dislocation	0
Endocarditis at follow-up	0
Follow-up time, days	350.8 ± 177.1

ICU, intensive care unit

## Discussion

In the present study, a total of 80 intermediate-risk patients presenting with moderate-to severe aortic valve disease underwent a conventional SAVR with sutureless aortic valve prosthesis. Of those, twelve patients were suffering from a pure severe aortic valve regurgitation. This study provides a number of interesting findings:

1. SU-AVR is a feasible and safe treatment option in patients presenting with the pure severe aortic valve regurgitation in isolated and combined procedures.
2. SU-AVR provides excellent hemodynamic performance with low transvalvular gradients at follow up and low CPB- and cross-clamp times.
3. The technical procedural success has been achieved in all patients. We report no paravalvular leakage or prosthesis dislocation at follow up.
4. We observed no cases of postoperative pacemaker implantation or stroke in our cohort.
5. None of the patients died at follow up.
6. Implementation of sutureless aortic valve prostheses is especially favorable for minimally invasive procedures.

Following the successes of transcatheter treatment methods in aortic valve replacement in high-risk patients, TAVR procedures have also been recently implemented as an alternative to conventional SAVR in intermediate- and low-risk patients [2]. Whilst transcatheter technologies have been very promising in patients presenting with moderate-to severe aortic valve disease, patients with the pure aortic regurgitation, with exception of some small studies on the off-label use, are officially not eligible for this treatment option [12]. SU-AVR has proven to be a feasible alternative to TAVR in patients with stenotic aortic valve disease, therefore we aimed to review our experience with the implementation of SU-AVR methods in patients with pure aortic valve regurgitation [9, 10, 13]. Given that TAVR procedure is strictly contraindicated in patients with isolated aortic regurgitation, SU-AVR could present the only minimally invasive treatment option for these patients.

The Perceval S aortic valve prosthesis is the only true sutureless prosthesis constructed of three leaflets of bovine pericardium mounted on a nitinol-stent. This prosthesis has been primarily created to be used in patients with severe aortic valve stenosis, and patients with a pure severe aortic regurgitation are considered ineligible for this procedure by the valve manufacturer. Similar to TAVR prostheses, Perceval S aortic valve is anchored into the aortic annulus using the radial forces of the nitinol stent. Therefore, the correct sizing of this valve prostheses is crucial to obtain the procedural success [6]. Too small prosthesis could easily dislocate from the left ventricular outflow tract and migrate, and a too large sized prosthesis could cause the incomplete deployment and a relevant paravalvular leakage. In all Perceval implantations in our cohort we used the previously described “Snugger method”, where the three placing sutures to guide the Perceval prosthesis into the aortic anulus are stabilized by the silicone snuggers. After the snuggers have been tightened, the valve prosthesis is deployed and the snuggers are carefully removed [6]. The correct sizing technique and the “Snugger-method” allowed us to achieve excellent technical results after Perceval valve in patients presenting with the stenotic aortic valve disease, as described previously, but also in our present cohort, in which we report of no cases of prosthesis dislocation or paravalvular leakage at follow-up.

Because of the ancoring of the prosthesis using only radial forces, there have been reported higher rates of permanent pacemaker implantation after SU-AVR. In our previous study cohort undergoing SU-AVR in patients mostly with stenotic aortic valve disease 3.1% of patients required a permanent pacemaker implantation and pure aortic regurgitation showed to be an independent predictive factor for postoperative pacemaker implantation [7]. In the SURD-IR Registry, Eusanio et al. report of a significant reduction of postoperative pacemaker implantation rates after completion of the learning curve [9]. Also, Mazine et al. in the Canadian multicenter study on sutureless valve implantation report of a rather high pacemaker implantation rate of 17% explaining this fact by possibly oversizing of the Perceval prostheses and therefore causing the mechanical damage to the conductance system [14]. In the German aortic valve registry, the authors describe a high rate of permanent pacemaker implantation of 13.7% in the Perceval cohort [15]. In the current study, we observed no cases of postoperative pacemaker implantation, which could be explained by the careful sizing and no need for extensive decalcification of the native aortic valve due to the isolated aortic regurgitation as a main pathology.

Sutureless valve prostheses have been constructed to effectively reduce the operating-, CPB-, and cross clamp time, which is especially important in elderly patients who have significantly lower tolerance for ischemia [16]. In our cohort we report a mean operating- and cross-clamp time of 127.25 ± 45.9 min and 40.33 ± 17.3 min respectively, which is lower than those reported in the German Aortic Valve Registry (GARY) [15]. Considering that more than half of our cohort underwent a concomitant procedure (58.3%), Perceval sutureless prosthesis allows a significant reduction of these operative parameters, which are known to have a great impact on the postprocedural outcomes. Furthermore, Perceval prosthesis offers an excellent hemodynamic performance also in patents with a pure aortic regurgitation with low mean transvalvular pressure gradients at 5.7 ± 1.5 mmHg. The gradients in our present cohort are significantly lower, than in our previous study, which can be explained, that in patients with pure AR larger prostheses sizes have been used compared to prostheses used in stenotic aortic valve disease and the additional element of the subvalvular stenosis in patients with hypertrophic heart disease due to the severe aortic valve stenosis is not relevant in the cohort with a pure aortic regurgitation [7]. We also did not observe any postoperative mortality during the follow up in out intermediate risk cohort [17].

In the SURD-IR registry, the authors report stroke rate of 2.8% in patients undergoing isolated SAVR with sutureless or rapid deployment valve prostheses [9]. In contrast to other studies, in out cohort we observed no cases of disabling stroke. This effect seems plausible, giving the noncalcified nature of the aortic valve disease in the present cohort. Furthermore, all the proximal CABG anastomoses were performed under cardiac arrest without partial clamping of the aorta to reduce the manipulation on the aorta and the dislocation risk of the Perceval prosthesis.

Minimal invasive aortic valve procedures have been introduced into the cardiac surgery in the early 90th and since then have gained popularity providing comparable to conventional SAVR postoperative results with less blood loss, better sternal stability, and superior cosmetic results [18–21]. In our cohort all the isolated SU-AVR were performed minimally invasive via J-sternotomy or right anterior lateral thoracotomy. Phan et al. compared both minimally invasive accesses and reported no significant differences in the postoperative outcomes after SAVR via J-Sternotomy and RALT [22]. Although the benefits of minimally invasive aortic valve surgery have been described my multiple trials, GARY Registry reports of a disappointingly low rate of minimally invasive SAVR in patients with isolated aortic valve disease [15]. This could supposable be due to the wrong perception of the technical difficulties surrounding minimally invasive procedures in general. Sutureless aortic valve prostheses have been constructed to simplify the implantation procedure and therefore are perfect for the utilization in minimally invasive aortic valve surgery, allowing to overcome its’ main limitation: the prolonged CPB- and cross-clamp time.

## Conclusion

Whilst indication for transcatheter aortic valve replacement has been extended towards intermediate- and low-risk patients, patients presenting with a pure aortic regurgitation still require a conventional SAVR. Similar to TAVR prostheses, sutureless aortic valve prostheses have been constructed to be utilized in patients presenting with stenotic aortic valve disease and implementation in severe aortic regurgitation is still contraindicated by the prosthesis manufacturer. Our center’s experience on SU-AVR shows the feasibility of the adoption of sutureless technologies into the aortic valve surgery due to the pure AR. Furthermore, besides the great technical success and excellent hemodynamics of the sutureless valves, SU-AVR in the severe AR offers a great opportunity of reducing the invasivity of the surgical procedure and potentially reducing hospital cost without compromising the postoperative outcomes and in-hospital length of stay.

### Study limitations

The retrospective non-randomized nature of the study coming from a single center with a limited number of patients may have an impact on the outcomes and the study power, and can leave room for bias. Further prospective studies on larger cohorts should be conducted to validate the safety and efficiency of this therapeutic alternative.

## Data Availability

The datasets used and analyzed during the current study are available from the corresponding author on reasonable requests.

## Abbreviations

AR: Aortic regurgitation
CABG: Coronary arterial bypass grafting
CPB: Cardiopulmonary bypass
IQR: Interquartile range
PFO: Patent foramen ovale
RALT: Right antero-lateral thoracotomy
SAVR: Surgical aortic valve replacement
SU-AVR: Sutureless aortic valve replacement
TAVR: Transcatheter aortic valve replacement
